# The Key Comorbidities in Patients with Rheumatoid Arthritis: A Narrative Review

**DOI:** 10.3390/jcm10030509

**Published:** 2021-02-01

**Authors:** Peter C. Taylor, Fabiola Atzeni, Alejandro Balsa, Laure Gossec, Ulf Müller-Ladner, Janet Pope

**Affiliations:** 1 Botnar Research Centre, Nuffield Department of Orthopaedics, Rheumatology and Musculoskeletal Sciences, University of Oxford, Oxford OX3 7LD, UK; 2 Rheumatology Unit, Department of Clinical and Experimental Medicine, University of Messina, 98122 Messina, Italy; fatzeni@unime.it; 3 Rheumatology Unit, Hospital Universitario La Paz, La Paz Institute for Health Research IdiPAZ, Universidad Autónoma de Madrid, Paseo de la Castellana, 261, 28046 Madrid, Spain; alejandro.balsa@salud.madrid.org; 4 Institut Pierre Louis d’Epidémiologie et de Santé Publique, Sorbonne Université, 75006 Paris, France; laure.gossec@aphp.fr; 5 Rheumatology Department, Pitié-Salpêtrière Hospital, AP-HP, Sorbonne Université, 75013 Paris, France; 6 Department of Rheumatology and Clinical Immunology, Justus Liebig University Gießen, Campus Kerckhoff, 61231 Bad Nauheim, Germany; U.Mueller-Ladner@kerckhoff-klinik.de; 7 St. Joseph’s Health Care, Schulich School of Medicine, University of Western Ontario, London, ON N6A 5C1, Canada; Janet.Pope@sjhc.london.on.ca

**Keywords:** rheumatoid arthritis, comorbidities, extra-articular manifestations, tumour necrosis factor, cardiovascular disease, infections, lymphoma, nonmelanoma skin cancers

## Abstract

Comorbidities in patients with rheumatoid arthritis (RA) are often associated with poor health outcomes and increased mortality. Treatment decisions should take into account these comorbidities due to known or suspected associations with certain drug classes. In clinical practice, it is critical to balance potential treatment benefit against the possible risks for comorbidities as well as the articular manifestations of RA. This review summarises the current literature relating to prevalence and risk factors for the important comorbidities of cardiovascular disease, infections, lymphomas and nonmelanoma skin cancers in patients with RA. The impact on patient outcomes and the interplay between these comorbidities and the therapeutic options currently available, including tumour necrosis factor inhibitors and newer biological therapies, are also explored. As newer RA therapies are developed, and patients gain wider and earlier access to advanced therapies, in part due to the emergence of biosimilars, it is important to consider the prevention or treatment of comorbidities as part of the overall management of RA.

## 1. Introduction

The relationship between rheumatoid arthritis (RA) and a wide range of comorbid conditions and extra-articular manifestations is well known [1,2,3]. The development of comorbidities is associated with poor health outcomes, including decreased function, reduced quality of life, and increased morbidity and mortality [1,2,4]. The identification of tumour necrosis factor (TNF) as a therapeutic target and its subsequent validation in clinical trials led to the approval of biologic TNF inhibitors for the treatment of RA over two decades ago, greatly improving the outlook for many patients [5,6,7]. The success of this therapeutic class was followed by the introduction of other biologic disease-modifying antirheumatic drugs (bDMARDs) and, more recently, small molecule targeted synthetic disease-modifying antirheumatic drugs (tsDMARDs) [7]. However, high costs of originator drugs have resulted in varying degrees of access restrictions across national health care economies [7,8]. Furthermore, although all targeted therapies have a broadly similar efficacy, they differ in mechanisms of action and associated target-related benefits and toxicities [9]. In the face of a wide range of available contemporary treatment options and management strategies for RA [10,11], careful consideration needs to be given to certain comorbidities with respect to treatment decisions [10,11,12]. The large international population-based, cross-sectional COMOrbidities in Rheumatoid Arthritis (COMORA) study evaluated the prevalence of comorbidities in 3920 patients with RA from 17 countries [1]. The most commonly observed comorbidities (past or current) were depression (15%), asthma (7%), cardiovascular (CV) events (myocardial infarction (MI), stroke; 6%), solid-organ malignancies (5%), and chronic obstructive pulmonary disease (4%) [1]. Results from the COMORA study demonstrated considerable intercountry variability for the prevalence of these comorbidities; for example, the prevalence of depression was 2% in Morocco compared with 33% in the USA [1]. For patients with RA, cardiovascular disease (CVD), infections and malignancies are important comorbidities as they may lead to an increased risk of mortality [1]. Furthermore, they are also impacted by at least one of the therapeutic options currently available for the treatment of RA [13,14,15] and may therefore affect treatment decisions in clinical practice. Notably, patients with congestive heart failure, active hepatitis B, or a history of other serious infections or malignancy are classified as high risk in the current American College of Rheumatology treatment guidelines for RA, and they have separate treatment recommendations [10].

In this review, the current literature is discussed with respect to the prevalence and risk factors for CVD, infections and selected malignancies (lymphoma and nonmelanoma skin cancers (NMSC)—basal cell carcinoma (BCC) and squamous cell carcinoma (SCC)). The impact on patient management and outcomes and associations with treatments employed in clinical practice is also explored. Although this review is not limited to TNF inhibitors, they are the focus of much of the discussion due to the availability of data. Moreover, due to the extensive clinical experience with these agents and the more recent impact of biosimilars on cost, they are expected to retain a major role in the treatment of RA in the foreseeable future [16,17].

## 2. Search Strategy

A narrative literature search was conducted on 8 September 2020 to address research questions related to the dominant comorbidities of CVD, infections, lymphoma and NMSC in patients with RA. For each of these comorbidities, the literature search aimed to identify publications relating to their prevalence and associated risk factors, their impact on clinical outcomes, health-related quality of life and patient management, and the effects of TNF inhibitors on these comorbidities.

The PubMed database was searched using a keyword search restricted to “Title Only” using terms relating to RA and cardiovascular (CV), infection, lymphoma, BCC or SCC. Search results were further refined by limiting the search using a MeSH Major Topic or keyword title search using search terms to identify publications discussing the prevalence of comorbidities, the factors contributing to comorbidities, the impact of comorbidities and the effect of TNF inhibitors on comorbidities. The complete search strings are provided in Table S1 in the supplementary materials. Only articles published within the past 5 years were included. Articles were reviewed for relevance based on the article title and abstract, with all article types taken into consideration if they included data relevant to the research questions.

To provide essential historical context to the current literature, several sections include references published before 2015. Additional references were identified through searching the bibliographies of retrieved articles, through a search of abstracts presented at the European League Against Rheumatism (EULAR) congress in 2019 and 2020, and through author suggestions.

## 3. CV Comorbidities in RA

### 3.1. Prevalence of CVD in Patients with RA

CVD is a major comorbidity affecting patients with RA and a leading cause of morbidity and mortality in this population [18]. Multiple recent studies have highlighted the extent to which patients with RA are affected by CVD, although comparisons between studies are often hindered by differences in the definition of events (Table 1). Patients with RA have been shown to be at higher risk of CVD compared with healthy controls. Using a large database of primary care patients across England, patients with early RA had a 33% higher CVD risk (composite endpoint of MI, stroke or heart failure) than matched controls without RA after adjustment for baseline differences including inflammatory markers, seropositivity and use of glucocorticoids (GCs) at diagnosis [19]. Similarly, in the CARdiovascular research and RhEumatoid arthritis (CARRÉ) long-term, prospective cohort study, patients with RA had a risk of CV events (coronary heart disease, cerebral arterial disease or peripheral arterial disease) that was almost double that of the general population. In the same study, the increased risk of CVD associated with RA was even higher than that associated with diabetes mellitus [20]. An increased risk of heart failure in patients with RA has also been highlighted recently in a systematic review that included over 5 million patients; in this study, the incidence of heart failure was almost two-fold higher in patients with RA than in matched controls [21]. It is important to note that the increased risk of CVD associated with RA may already be present at an early stage of the disease, with an excess of stroke and heart failure reported prior to RA diagnosis compared with matched controls in England [19].

Imaging studies have also demonstrated the increased risk of CV in patients with RA. A study of patients referred for computed tomography angiography due to chest pain reported a higher prevalence of coronary artery calcification in RA patients than in controls [29]. The strongest associations were reported in seropositive RA patients and in patients requiring treatment with GCs for relapse or flare; treatment with conventional synthetic disease-modifying antirheumatic drugs (csDMARDs), but not bDMARDs, was also associated with a tendency for increased risk of obstructive coronary artery disease (CAD). Consistent with these findings, a recent meta-analysis of computed tomography (CT) studies reported that asymptomatic CAD is more prevalent in patients with RA than in controls [30]. Although the evidence was limited, it was also noted that patients with RA had a higher prevalence of moderate-to-severe CAD and more multivessel CAD [30]. CV abnormalities in patients without overt cardiac disease have also been detected by echocardiography in patients with RA. In a prospective study in Italy, a five-fold increased risk of abnormal changes to the structure and function of the left ventricle was observed in patients with RA compared with matched controls [31]. In this study, left ventricular strain was independently associated with a 2.4-fold increased risk of all-cause hospitalisation and a 6.6-fold increased risk of CV-related hospitalisation.

### 3.2. Impact of CV Comorbidities in Patients with RA

Patients with comorbid CVD and RA tend to have worse long-term health outcomes than patients with CVD alone. In a large population cohort study in Denmark of patients undergoing coronary angiography, the 10-year risk of MI was higher in patients with RA and coincident CAD compared with non-RA patients with CAD (12.2% vs. 9.9%). Similar associations were observed for major adverse CV events (MACE) and all-cause mortality [32]. Worse outcomes were also noted in a recent meta-analysis, which demonstrated that patients with co-existing CAD and RA had significantly increased risks of all-cause mortality, cardiac death and congestive heart failure compared with CAD patients without RA. In patients who underwent percutaneous coronary intervention, all-cause mortality rates remained significantly higher in patients with RA and CAD than in non-RA patients with CAD [33]. Similarly, in a large cohort study of patients who experienced an acute coronary syndrome (ACS) event, patients with RA had a 27% and 50% increased risk of ACS recurrence or mortality, respectively, after a mean 2-year follow-up compared with controls, which remained statistically significant even after adjustment for baseline comorbidities. This increased risk could not be explained by differences in the use of standard-of-care secondary preventative drugs [34].

The presence of RA can also have a negative impact on short-term outcomes following CV events. In a recent database study in Taiwan, patients with RA had a higher risk of in-hospital mortality after acute MI, intracranial haemorrhage or ischaemic stroke compared with patients without RA [35]. Similarly, a large Swedish study demonstrated that mortality rates within 1 week and 1 month following an ACS event were significantly higher in patients with RA compared with a matched cohort from the general population, even after adjustment for age, sex, pre-existing comorbidities and pharmacotherapies, and ACS type. Patients with RA also had higher troponin levels, and higher frequencies of in-hospital complications and ST-segment elevation MIs compared with the control cohort [36]. In contrast, a small study in Israel found that the outcome and prognosis of patients with ACS were not affected by the coexistence of inflammatory rheumatic diseases. The authors of this study argued that excess mortality in patients with rheumatic diseases reflects the higher prevalence of CVD in this population rather than worse outcomes for CV events [37]. However, it should be noted that this study included only 20 patients with ACS and inflammatory rheumatic conditions, of whom only 11 had RA.

### 3.3. Risk Factors for CVD in Patients with RA

The increased CVD risk observed in patients with RA is likely to be multifactorial, reflecting an increased prevalence of traditional CVD risk factors, the impact of systemic inflammation and potential side effects from medications used to treat RA.

The burden of traditional risk factors for CVD among patients with RA has been extensively documented, with multiple large studies reporting a high prevalence of current tobacco use (ranging from 19 to 29%), hypertension (19 to 61%), diabetes mellitus (5 to 14%) and hyperlipidaemia (10 to 32%) [1,3,19,20,24,28]. In addition, many patients were reported to be overweight or obese (mean body mass index (BMI) ranged from 27 to 29 kg/m^2^) [19,20,24,38]. In the COMORA study, around half of the enrolled patients were overweight or obese (51%), and almost half (43%) were considered to have a high 10-year risk of CVD based on the Framingham score [1]. Compared with the general population, the prevalence of traditional CVD risk factors is often higher amongst patients with RA. For example, in a large study of primary care patients in England, patients with RA had a higher BMI and were significantly more likely to have diabetes and to be current or former smokers than age- and gender-matched controls without RA [19]. A similar pattern was reported in the CARRÉ study, with patients with RA having a significantly higher prevalence of hypertension and tobacco use than a control population [20]. Interestingly, in both studies, total and low-density lipoprotein (LDL) cholesterol levels were lower in patients with RA than in controls [19,20].

To date, numerous studies have shown that traditional risk factors contribute to the increased CVD risk faced by patients with RA. In a large, international cohort study, traditional risk factors were responsible for 49% of the CV risk in RA patients, with current smoking status and hypertension being major modifiable risk factors [24]. Similar conclusions were drawn by Nikiphorou et al., who reported that current smoking, BMI and diabetes were associated with a higher rate of CVD among patients with RA [19]. At a subclinical level, traditional risk factors (age, mean arterial pressure and diabetes) have also been shown to be independently associated with worsening atherosclerosis in patients with RA who had not experienced previous CV events [39].

**Figure 1:**
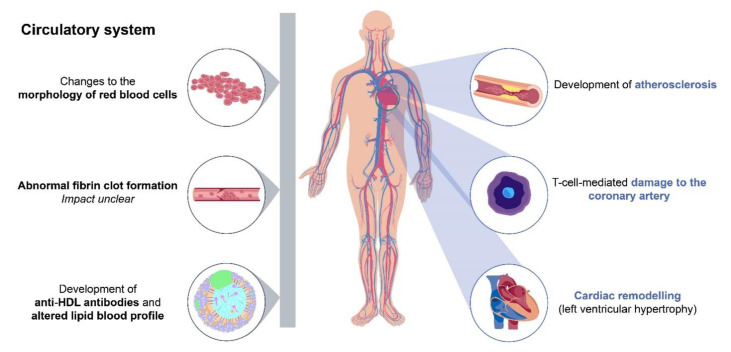
Chronic inflammation in rheumatoid arthritis as a risk factor for cardiovascular disease. The proinflammatory mechanisms underlying RA may contribute to the development of atherosclerosis [45,46,47], promotion of cardiac remodelling [48], alterations in lipid blood profiles [49] and changes to the morphology of red blood cells [50]. RA can be exacerbated by cytomegalovirus infection and is linked to coronary artery damage via cytotoxic T-cells [51]. The autoimmune mechanisms that drive RA have been linked to abnormal fibrin clot formation and increased CVD risk; however, the extent to which this is affected in RA remains unclear [52]. Grey bar indicates nonlocalised effects. Abbreviations: CVD, cardiovascular disease; HDL, high-density lipoprotein; RA, rheumatoid arthritis.

The presence of traditional risk factors does not fully account for the increased CVD risk in patients with RA, with many studies reporting that a considerable residual risk remains even after adjustment for traditional risk factors [19,20,24]. Systemic inflammation may, at least in part, explain the remaining risk, and this theory is supported by a multitude of studies demonstrating an association between high RA disease activity and increased CVD risk (Table 2) [24,28,39,40,41,42,43,44]. The relationship between chronic inflammation and CVD remains to be fully delineated, but it has been reported that the proinflammatory mechanisms underlying the pathogenesis of RA may contribute to the development of atherosclerosis [45,46,47], promotion of cardiac remodelling [48], alterations in lipid blood profiles [49] and changes to the morphology of red blood cells [50] (Figure 1). RA-related inflammation can also be exacerbated by cytomegalovirus infection and is linked to coronary artery damage through the actions of a population of cytotoxic T-cells (reviewed in detail by Broadley et al. [51]). Autoimmune mechanisms that drive RA progression have also been linked to abnormal fibrin clot formation and increased CVD risk. However, the extent to which haemostasis is affected in RA remains unclear (reviewed in detail by Bezuidenhout et al. [52]).

Targeted CVD risk management is an important part of the overall clinical management of patients with inflammatory joint disorders, including RA [53]. Guidance based on expert opinion and scientific evidence was issued by EULAR in 2017 and includes the importance of optimal control of disease activity, CVD risk assessment every 5 years and lifestyle recommendations [53]. CVD risk prediction models should incorporate a multiplication factor of 1.5 for patients with RA (if not already included) and screening for asymptomatic atherosclerotic plaques by carotid ultrasound should be considered; however, this has not yet been assessed in a clinical setting [53]. In terms of treatment, nonsteroidal anti-inflammatory drugs (NSAIDs) should be used with caution in patients with documented CVD or with CVD risk factors, and the dose of GCs should be kept to a minimum for prolonged treatments [53]. The guidelines also emphasised the important role of the rheumatologist in CVD risk management [53]. Fortunately, physicians appear to be aware of the need to monitor CVD risk in patients with active RA: a study of 14,503 patients in world-wide data from the SUrvey of cardiovascular disease Risk Factor management in Rheumatoid Arthritis (SURF-RA) database demonstrated that positivity for rheumatoid factor and anticitrullinated protein antibodies, longer disease duration and higher disease activity (measured by Disease Activity Score 28 joint count-C reactive protein (CRP)) was associated with a higher likelihood of lipid and blood pressure assessments [54].

In order to improve the management of comorbidities in chronic inflammatory rheumatic diseases in daily practice, an initiative supported by EULAR aimed to standardise reporting and screening of comorbidities [12]. For CVD, this included the use of a standardised form for reporting a history of ischaemic CV diseases, risk factors and CVD-related treatments [12].

### 3.4. Effect of RA Treatments on CV Risk

In addition to traditional CV risk factors and inflammatory processes, CVD risk may also be altered by some of the common medications used for the treatment of RA. For example, corticosteroids and NSAIDs, particularly COX-2 inhibitors, are generally associated with an increase in CVD risk in patients with RA (reviewed by Jagpal et al. [55] and DiMizio et al. [18]). Conversely, nonbiologic DMARDs, such as methotrexate, are associated with an improved CVD risk [56].

As chronic inflammation is considered to be a modifiable risk factor for the development of CVD, targeting systemic inflammation with TNF inhibitors has the potential to reduce CVD risk in patients with RA [57]. This is supported by a meta-analysis from 2015, which demonstrated that TNF inhibitors and methotrexate significantly reduced the risk of CV events compared with no treatment; however, treatment with NSAIDs or corticosteroids led to an increase in risk [58]. Similarly, a large study of patients with RA recruited to the British Society for Rheumatology Biologics Register for Rheumatoid Arthritis (BSRBR-RA) demonstrated that treatment with TNF inhibitors significantly reduced the risk of MI compared with csDMARDs, although no differences in MI severity or mortality were observed between treatment groups [59]. The benefits of TNF inhibitors and other biologics were also demonstrated in a prospective cohort study from Australia, which concluded that, after adjustment for a number of risk factors, the risk of CV events was significantly reduced following the use of these agents in patients with RA, psoriatic arthritis (PsA) or ankylosing spondylitis (AS) [60]. Length of treatment may also have an impact on CV risk, with a large retrospective insurance claims database study in the USA reporting a positive correlation between duration of treatment with TNF inhibitors and reduction in CV risk. This study concluded that cumulative use of 1, 2 or 3 years of anti-TNF therapy is expected to reduce CV risk by 21%, 38% and 51%, respectively, compared with non-use [61].

Consistent with the reduced risk of clinical events, treatment with TNF inhibitors may also lead to improvements in CV abnormalities detected by imaging. For example, patients with early RA and no CVD history have been shown to have abnormal vascular stiffness, evidence of diffuse myocardial fibrosis and reduced left ventricular mass compared with controls. Treatment with methotrexate and etanercept (either concomitantly or following a step-up strategy) resulted in significant improvements in vascular stiffness after 1 year [62]. Similarly, patients with RA have been reported to have impaired left ventricle longitudinal strain, which improved after treatment with TNF inhibitors [63].

The benefits of TNF inhibitors on CVD risk correlate with their impact on RA disease control, with data from the Swedish biologics register demonstrating that the 1-year risk of ACS for patients with a good EULAR response was approximately half that of patients with no EULAR response [64]. Similarly, improvements in the apolipoprotein profile, a biomarker of CVD risk, were observed in patients with RA who exhibited a good or moderate EULAR response to etanercept but not in EULAR nonresponders [65]. The relationship between RA disease control and the impact of TNF inhibitors on CV risk suggests that these agents may exert their effects through reducing systemic inflammation rather than by modifying traditional CV risk factors. This is supported by a phase IV, randomised, double-blind, placebo-controlled study, which demonstrated that etanercept did not affect levels of traditional metabolic risk factors (including glucose, insulin, lipid and apolipoprotein parameters) despite reducing RA severity, as indicated by decreases in CRP [66]. Additional support for this theory includes a molecular profiling study, which demonstrated that patients with RA at the greatest risk of CVD expressed high levels of biomolecules, which are known to be mediators of autoimmunity, inflammation and oxidative damage. Importantly, bDMARDs, such as TNF inhibitors and cluster of differentiation 20 (CD20) inhibitors, were shown to re-establish normal levels of these circulating biomolecules and, therefore, reduce CVD risk [67].

The impact of newer classes of therapy on CVD risk is also of considerable interest for the management of patients with RA. Concerns have previously been raised regarding lipid elevations associated with the interleukin-6 inhibitor tocilizumab, including significantly increased levels of total and LDL cholesterol [68,69,70]. However, this does not appear to translate to increased clinical risks, with a number of studies showing no significant differences between TNF inhibitors and tocilizumab in terms of CV risk [71,72,73]. This included the ENTRACTE randomised controlled trial (RCT), which demonstrated no significant differences in the risk of MACE between tocilizumab and etanercept in a large population of patients with RA and at least one CV risk factor [71]. Conversely, there is some limited evidence that tocilizumab may offer CV benefits compared with TNF inhibitors, including improvements in lipoprotein markers [74,75] and a lower risk of MACE [76]. Similarly, a large study utilising data from Taiwan’s National Health Insurance claims database reported that TNF inhibitor nonresponders who received tocilizumab had a lower risk of CV events compared with patients who received rituximab [77]. The disconnect between clinical CVD risk and elevated lipids remains to be fully elucidated but may relate to protective changes to other surrogates of CV risk following treatment with tocilizumab. For example, in the MEASURE and ADACTA trials, tocilizumab treatment altered high-density lipoprotein particles towards an anti-inflammatory composition (reduced serum amyloid A content) and induced a significant reduction in secretory phospholipase A2-IIA, lipoprotein(a), fibrinogen and D-dimers [68,70]. Abatacept, which inhibits the activation of T-cells, has also been associated with modest decreases in the risk of CV events compared with TNF inhibitors [78,79] and rituximab [77].

Janus kinase (JAK) inhibitors and, in particular, tofacitinib, have been associated with an increased risk of venous thromboembolisms (VTEs) in postmarketing surveillance studies [80,81]. However, it should be noted that this increased risk was only observed when tofacitinib was used at a dose of 10 mg twice a day, which is higher than the dose approved for RA in most countries [82,83]. Moreover, a recent meta-analysis of 26 RCTs, comprising 11,799 patients, indicated that treatment with JAK inhibitors as a class or as individual therapies did not affect the risk of VTEs, CV events or MACE in patients with RA, at least in the short term [84].

## 4. Infections in Patients with RA

### 4.1. Prevalence of Infections in Patients with RA

The risk of infections and serious infections (SI) has long been known to be increased in patients with RA [85,86]. Historically, a longitudinal, retrospective cohort study of US-based patients with RA and a disease onset between 1955 and 1994 found the rate of infections requiring hospitalisation among patients with RA was almost twice that of the age- and sex‑matched patients without RA [85]. More recently, a longitudinal, prospective, real-world study of patients included in the FORWARD database (the largest patient-reported research data bank for rheumatic disorders in the US [87]) from 2001 to 2016 reported that the risk of SI and joint infections was 70% higher in patients with RA (*n* = 20,361) compared with a matched cohort of patients with noninflammatory rheumatic and musculoskeletal diseases (*n* = 6176; hazard ratio (HR): 1.7; 95% confidence interval (CI): 1.5–1.8) [88]. Subsequent data from the FORWARD database demonstrated that 5.9% of patients with RA treated with TNF inhibitors, non‑TNF inhibitors, bDMARDs (abatacept, rituximab, tocilizumab and anakinra) or csDMARDs (*n* = 11,623) experienced an SI during 27,552 patient-years of follow-up [89]. Studies from other parts of the world reinforce the association between RA and the increased burden of infection, with studies from the UK and India reporting that between 3% and 8% of patients with RA experience infections (Table 3) [90,91]. A diagnosis of RA may also increase infection risk in a surgical setting, with a retrospective, US-based case–control study (*n* = 2212) reporting that RA was associated with a 47% increased risk of postoperative infection following total joint arthroplasty vs. patients with osteoarthritis (odds ratio (OR): 1.47; *p* = 0.031) [92]. Despite the wealth of evidence supporting the increased susceptibility of patients with RA, infections are suboptimally prevented, screened for and managed [12].

Patients with RA are particularly susceptible to bacterial infections (vs. viral or fungal) [88], and those most frequently reported include respiratory infections [88,93], urinary tract infections (UTI) [93], *mycobacterium tuberculosis* infection (TB) [96] and sepsis [88]. Interestingly, patients with autoimmune inflammatory rheumatic diseases are at an increased risk of contracting a number of vaccine-preventable infections (influenza, pneumococcal, disease, herpes zoster (HZ) and human papillomavirus) [97]. Given this, vaccinations are strongly recommended for this patient population; however, vaccination uptake is low [91,98]. For instance, a UK-based, real-world study of patients with RA demonstrated that over 85% of patients were vaccinated against influenza, but only 44% were vaccinated against pneumococcus [91]. To increase vaccination uptake, the authors advised that collaborative approaches between primary and secondary care are required and stressed the need to overcome potential negative perceptions related to vaccine safety [91].

Preliminary data on COVID-19 in patients with RA have been emerging throughout 2020, and although data are limited at the time of writing in December 2020, most data indicate that patients with RA are not at an increased risk of contracting COVID-19 compared with the general population [99,100]. A systematic review of eight observational cohort studies, comprising 6095 patients with rheumatic disease, indicated that patients were not particularly susceptible to COVID-19. The majority of those infected experienced a mild clinical course, and there was a low level of fatalities [100]. Two survey studies of patients with rheumatological diseases also demonstrated that the incidence of COVID-19 and associated symptoms were consistent with the general population [101,102], although, in a study from the Global Rheumatology Alliance registry, patients with rheumatic disease (*n* = 600) and comorbidities, such as hypertension, CVD and diabetes, had higher odds of hospitalisation due to COVID-19 [103]. Discordantly, a large meta-analysis of 62 observational studies (*n* = 319,025) from Akiyama et al. demonstrated that patients with autoimmune disease had a higher prevalence of COVID-19 compared with the general population [104]. Akiyama et al. also evaluated the results from six case–control studies that demonstrated that the risk of COVID-19 in patients with RA was significantly higher than in the control groups (OR: 1.6; 95% CI: 1.1–2.3; *p* = 0.008) [104]. When interpreting results, it is important to consider that observational data may underestimate infection risk, as patients will often be aware of the risks inherent to their disease and may have adapted their lifestyle to reduce the probability of contracting COVID-19 [105].

### 4.2. Impact of Infections in Patients with RA

The presence of autoimmune diseases, including RA, appears to increase SI-related morbidity and mortality; however, data are limited [106,107]. A study from the German Rheumatoid Arthritis: Observation of Biologic Therapy (RABBIT) biologics register demonstrated that SI escalated to sepsis or preceded the patient’s death for one in five patients with RA [95]. A recent study from Germany also reported that mortality due to sepsis in patients admitted to intensive care was higher in patients with RA compared with age- and sex-matched controls (*p* = 0.0016) [106]. Additionally, a study in Brazil showed that patients with RA and a primary dengue infection had higher rates of hospitalisation and death compared with patients with RA without dengue infection [108]. Conversely, in a population-based cohort study in Denmark of patients hospitalised for pneumonia, the overall 30-day or 90-day mortality rates were not higher for patients with RA compared with patients without RA [107]. The association between SI and RA disease outcome is unclear. In a retrospective analysis of 370 patients with RA, multiple SIs that required hospitalisation were associated with an advanced physical disability and radiological joint destruction [109]. The results highlight that the influence of RA on SI outcomes, and the influence of SI on RA outcomes, is complex and warrants further investigation.

### 4.3. Risk Factors for Infections in Patients with RA

The increased infection risk in patients with RA has been attributed to the impact of immunocompromising comorbid conditions, the underlying immune dysregulation associated with the disease itself and the sequelae of immunosuppressive therapy [86,89]. However, it is difficult to quantify the specific contribution of each of these factors.

#### 4.3.1. Comorbidities as a Risk Factor for Infection in Patients with RA

A number of patient demographics and comorbidities have been shown to be predictive of the observed increased risk of infection in patients with RA [86]. Factors associated with an increased infection risk include male sex [92,93,96], older age [90,93,95,96,110,111], longer RA disease duration [90,93], a history of SIs [88], lower education [90], family income [90], rural residency [88], geographical location [111], poor nutritional status [112] and higher BMI [111]/obesity [92]. Significantly increased infection risks have been described for patients with comorbid conditions including renal dysfunction [93,95,113], pulmonary disease [89], underlying lung disease [93,112] and diabetes [88,92,111]. The number of comorbidities has been observed to correlate with an increased risk of infection [88,89].

#### 4.3.2. Disease Activity as a Risk Factor for Infection in Patients with RA

There is evidence suggesting that the inflammatory processes underlying RA may also drive the development of infections. This is supported by the numerous studies demonstrating the association of active RA with an increased risk of infection. For instance, in a retrospective analysis of patients enrolled in the US-based CORRONA registry, the adjusted risk of SI was 69% higher in patients with sustained low disease activity compared with those in sustained remission (adjusted incidence rate (IR) ratios: 1.7; 95% CI: 1.3–2.2). In patients with moderate-to-high disease activity, the risk of SI was more than double that observed for patients in sustained remission (IR/100 patient-years: 2.5; 95% CI: 2.2–2.8 vs. IR/100 patient-years: 1.0; 95% CI: 0.9–1.3) [114]. In addition, the aforementioned population-based cohort study in Denmark of patients hospitalised for pneumonia demonstrated that high RA disease activity prior to hospitalisation was associated with a five-fold increased risk of 90-day mortality compared with patients with low RA activity, although overall mortality rates were not higher for patients with RA vs. patients without RA (19.9% and 18.9%, respectively) [107]. A study of the FORWARD database reported that compared with patients with noninflammatory rheumatic and musculoskeletal diseases, patients with RA with low disease activity, or in remission, had a similar SI risk; however, patients with moderate and high RA disease activity had a significantly increased SI risk [88]. Authors of these studies discussed the clinical relevance of their results and suggested that for patients with high RA disease activity, the treatment goal should be to obtain low-grade disease activity or remission as this may result in improved outcomes for patients with SI [88,107]. Accortt et al., authors of the study of the CORRONA registry, even suggested that their results may incentivise patients and health care providers to strive for remission rather than low disease activity [114]. In contradiction, however, Mehta et al. cautioned that while low disease activity/remission is an attractive target, clinicians should weigh the potential SI risk associated with aggressive treatment strategies in patients with RA while targeting and sustaining remission or low disease activity [88].

#### 4.3.3. Treatment Regimens and the Risk of Infection in Patients with RA

Regimens used frequently in the treatment of RA, including GCs and DMARDs, have been associated with an increased risk of infection or the reactivation of latent infections (Table 4).

##### GCs Increase the Risk of Infection in Patients with RA

GCs are effective and widely used in RA, although the benefit-to-risk ratio of this treatment remains precarious [118]. The role of GCs in increasing patient susceptibility to SIs and the associated mortality is supported by numerous studies [88,93,95]. For example, in a study of the FORWARD database, the current use of GCs was the strongest predictor of SI. Additionally, when the HR was adjusted to account for GC use, the excess risk of SI for patients with RA, compared with a matched cohort of patients with noninflammatory rheumatic and musculoskeletal diseases, was attenuated from 70% to 30% (HR: 1.3; 95% CI: 1.2–1.5) [88].

##### csDMARDs and the Risk of Infection in Patients with RA

Evidence suggests that csDMARDs, such as methotrexate, confer no increased risk for SI or SI-associated mortality in patients with RA [107]. Indeed, the addition of methotrexate to a bDMARD regimen did not increase the risk of SI compared with bDMARD monotherapy in a meta-analysis of patients with RA [119]. These conclusions, however, differ from those from a Japanese cohort study, which identified the use of methotrexate as a risk factor for *Pneumocystis* pneumonia, although methotrexate use was negatively associated with the incidence of all infections [93].

##### bDMARDs and the Risk of Infection in Patients with RA

The risk of infection is generally increased with bDMARDs compared with csDMARDs [89,116]. In a large meta-analysis of 106 trials published between 1992 and 2014, an increase in the risk of SI was reported for patients treated with standard- or high-dose bDMARDs compared with csDMARDs; however, no increase in risk was noted for patients treated with low-dose bDMARDs [116]. Correspondingly, in a 10-year retrospective review in patients with RA in Western Australia, the mean duration of bDMARD use correlated with a greater risk of SI (*p* = 0.04) [110]. These conclusions differ from those reported by Richter et al. that patients treated with bDMARDs had a significantly lower risk of developing sepsis (OR: 0.6; 95% CI: 0.4–0.8) and had a significantly lower mortality risk than those treated with csDMARDs. Richter et al. proposed that successful immunosuppression may prevent escalation to sepsis but noted that further investigation is required [95].

##### TNF Inhibitors and the Risk of Infection in Patients with RA

The increased risk of infections is one of the most important considerations when prescribing TNF inhibitors, although the magnitude of this risk is a topic of debate [120]. Results from a study of the FORWARD database demonstrated that the SI incidence rate per 1000 patient-years was increased in patients receiving TNF inhibitors (26.9; 95% CI: 24.5–29.6) or non-TNF-inhibitor bDMARDs (23.3; 95% CI: 19.0–28.5) vs. csDMARDs (22.4; 95% CI: 19.2–26.1) (Table 4) [89]. A meta-analysis of 71 RCTs and seven open-label extension studies in patients with RA, PsA and AS in 2014 found significant increases in the occurrence of any infections, SI and TB associated with TNF inhibitor treatment (20%, 40% and 250%, respectively) [117]. These findings, however, differ from those from the BSRBR-RA, where it was reported that patients with RA who discontinued TNF inhibitors 60 days after an SI were at higher risk of SI recurrence compared with patients who remained on TNF inhibitor therapy or switched to a different biologic class [121]. There are a number of explanations for this discrepant observation; it is possible that there is a clinical advantage of continuing biologic immunosuppression following an SI, although the authors advise against inferring a causal link. It could be that these data reflect a channelling bias whereby the “fittest” patients with the lowest baseline risk of experiencing recurrent SI were preferentially selected to restart biologics post-SI [121]. Overall, data suggest that TNF inhibitor use is associated with an increased risk of infection in patients with RA.

Differences in the infection risk between individual TNF inhibitors is difficult to determine due to the scarcity of head-to-head RCTs and the difficulties associated with cross-trial comparisons. Data from a US RA registry study demonstrated that there were no differences in the risk of SI between certolizumab pegol compared with other TNF inhibitors (etanercept, adalimumab, golimumab or infliximab) [122]. Contrastingly, a meta-analysis of 12 observational studies reported a lower incidence of SI in patients receiving etanercept compared with the TNF inhibitors infliximab or adalimumab (relative risk (RR): 0.6; 95% CI: 0.4–1.0; *p* = 0.04), although the authors noted that heterogeneity between the studies was high and likely due to differences in study designs and clinical features and, therefore, cautioned against drawing conclusions [120]. Data from a 10-year retrospective review demonstrated that the lowest rate of SI was observed in those receiving treatment with adalimumab (5.3/100 person-years) and it was highest with infliximab (34.5/100 person-years) vs. other bDMARDs [110].

Multiple studies have reported no significant differences in the risk of infection between TNF inhibitors when compared with other biologics and drug classes. For instance, a study of the BSRBR-RA reported that after adjusting for confounding variables, there were no differences between a number of different biologics (all biologics, TNF inhibitors, rituximab and tocilizumab) in the risk of SIs for patients with RA [96]. A study of the FORWARD database also demonstrated that the risk of SI among patients with RA treated with TNF inhibitors was not significantly different from that observed with other non-TNF-inhibitor bDMARDs [89]. However, other studies have reported specific differences. A study of claims data from US Medicare reported that the risk of serious bacterial infections, diverticulitis, and skin and soft tissue infections was lower in patients receiving TNF inhibitors compared with those receiving tocilizumab; however, no differences in the risk of SI overall were reported [123]. Discordantly, in Rutherford et al.’s study of the BSRBR-RA, patients with RA treated with etanercept had a lower risk of SI vs. patients treated with tocilizumab (HR: 1.2; 95% CI: 1.0–1.5) [94]. It is worth noting that channelling bias can present a challenge with such trial designs, particularly when some treatments are administered in a first-line setting and others are more routinely administered later in the treatment pathway. However, to account for this, Rutherford et al. excluded biologic-naïve patients in a sensitivity analysis and the results remained significant. Rutherford et al. also reported that prior to adjusting for confounding, etanercept had a lower incidence of infection than rituximab; however, once adjusted, the difference lost significance, suggesting that patient factors rather than the regimen itself may be responsible for the difference [94]. A separate study of the BSRBR-RA from the same group found that the incidence of pneumocystis was significantly lower following treatment with TNF inhibitors vs. rituximab (adjusted HR: 3.2; 95% CI: 1.4–7.5) [96].

There are also limited data suggesting that patients treated with TNF inhibitors may have an increased risk of hospitalisation due to infection compared with patients treated with abatacept. Evidence from a propensity-score-matched study of 11,248 pairs of patients with RA included in the MarketScan database demonstrated increased rates of hospitalisation due to infection in patients treated with TNF inhibitors (as a class) compared with those treated with abatacept (HR: 0.8; 95% CI: 0.6–1.0) [124]. Of note, hospitalisation rates due to infection continued to be higher when infliximab was compared with abatacept (HR: 0.6; 95% CI: 0.5–0.9), although no significant differences were reported when etanercept or adalimumab was compared with abatacept [124]. Conversely, a cohort study analysing Medicare data reported that patients treated with etanercept, infliximab or rituximab had a significantly higher 1-year risk of hospitalisation due to infection than patients treated with abatacept (adjusted HRs: 1.2, 1.4 and 1.4, respectively). In contrast, no significant differences were reported for adalimumab, certolizumab, golimumab or tocilizumab when compared with abatacept, after adjusting for potential confounding factors (adjusted HRs for all: 1.1) [125].

##### tsDMARDs and the Risk of Infection in Patients with RA

The more recently introduced tsDMARDs, such as JAK inhibitors, have an unclear association with infection risk. Some studies report that JAK inhibitors have a similar risk for infections as bDMARDs; for example, the SI rate for tofacitinib in RA clinical trials was approximately 3 per 100 patient-years [115]. Moreover, in a systematic analysis of 21 studies of tofacitinib, baricitinib and upadacitinib, the IRs for SI were 2.0 (95% CI: 1.4–2.7), 3.2 (2.1–4.6) and 3.0 (1.0–7.0), respectively [126]. Discordantly, an on-going, open-label, postmarketing clinical study evaluating the safety of tofacitinib compared with TNF inhibitors (etanercept or adalimumab) in patients with RA demonstrated that tofacitinib was associated with increased mortality, which was partially attributable to the increased SI risk, vs. TNF inhibitors [81]. Based on this observation, the European Medicines Agency recommends that patients aged over 65 years should only be treated with tofacitinib when there is no suitable alternative treatment [82]. It is evident that the general SI risk with JAK inhibitors is not yet fully understood, and it will be interesting to see what further research reveals.

A significant association between HZ risk and treatment with JAK inhibitors has been reported [126], a finding supported by a study of the German registry, RABBIT, which also demonstrated an increased risk for HZ following tsDMARD treatment (HR: 3.6; 95% CI: 2.3–5.4) [127]. These results also agree with findings from a pooled analysis with a total of 8.5 years of tofacitinib exposure, where SIs were observed in 8.5% of patients at a rate of 2.7 per 100 patient-years, which did not increase during the follow-up [111]. The most common types of infection from the pooled analysis were pneumonia, HZ, UTI and cellulitis; HZ occurred at a rate of 3.9 (95% CI: 3.6–4.2) per 100 patient-years [111]. These results support the HZ vaccination of patients with RA, particularly if patients are to receive JAK inhibitors.

##### RA Treatment Regimens and the Risk of Reactivating Latent Infections

TNF inhibitors have been associated with an increased risk of reactivating latent infections. In a meta-analysis of randomised studies, exposure to TNF inhibitors was associated with a three-fold increase in the risk of TB reactivation compared with placebo or no treatment (fixed-effects model, OR: 3.5, 95% CI: 1.6–7.9; random-effects model, OR: 3.3, 95% CI: 1.5–7.3) [117]. The risk of TB infection and reactivation also appears lower in etanercept-treated patients compared with those treated with infliximab or adalimumab, as observed in several studies [96,120,128]. In patients with risk factors for TB, etanercept may, therefore, be the most suitable TNF inhibitor of choice. Moreover, the incidence of TB was significantly higher among those treated with TNF inhibitors compared with rituximab [96]. TNF inhibitors are also reported to increase the likelihood of hepatitis consequent to hepatitis B virus (HBV) reactivation; however, data on the specific increased risks vary between TNF inhibitors, from 12.5% to 62.5% [129,130,131]. The potential for reactivation of HBV in patients treated with TNF inhibitors has been compared with non-TNF-inhibitor bDMARD therapy in a retrospective cohort study, which demonstrated no reactivation of HBV in the group receiving TNF inhibitors, compared with two cases in the group that received bDMARDs; however, this difference was not significant (*p* = 0.226) [132].

Other regimens used frequently in the treatment of RA have also been associated with an increased risk of reactivating latent infections, including HZ [133,134] and HBV [135]. Patients receiving GC and/or csDMARD therapy have an established increased risk of HBV reactivation, and bDMARD therapy is also emerging as a potential driver for hepatitis reactivation [135]. This is evident by the boxed warning regarding HBV reactivation that has been added to the prescribing information for the bDMARD rituximab [136]. Indeed, the Centers for Disease Control and Prevention Guidelines recommend screening for HBV prior to initiating immunosuppressive therapies [137]. Moreover, the EULAR and Italian consensus guidelines also state that patients with rheumatic diseases, classified as “at risk”, should be vaccinated against HBV [98,138].

The impact of HBV infection on clinical response to bDMARDs is less well documented. A retrospective analysis of occult HBV infection and the impact it has on drug survival in patients with RA was conducted using the Biologic Apulian Registry [139]. This study revealed that patients with occult HBV (*n* = 110) were significantly more likely to receive tocilizumab or abatacept as their first DMARD compared with those who were occult-HBV-negative (*n* = 376). Patients with occult HBV had significantly lower drug survival rates compared with patients without occult HBV. Consequently, patients with occult HBV had higher levels of discontinuation due to drug ineffectiveness (occult-HBV-positive, 66.4% vs. occult-HBV-negative, 75.3%; long rank: 7.9; *p* = 0.005) [139]. This finding further emphasises the importance of screening and monitoring patients for certain infections prior to initiating, and during, treatment with immunosuppressive therapy.

##### The Impact of RA Treatment Regimens on the Severity of COVID-19 Infection

Studies on the impact of RA therapies on the severity of COVID-19 infection are limited, and professional organisations, such as EULAR, have described themselves as “flying blind” due to the novelty of the pandemic and the lack of sound evidence [140]. Thus far, data, however limited, indicate that GC usage may increase the risk of hospitalisation in infected patients with rheumatic disease when compared with other treatment regimens [141]. For example, a prospective study on the impact of COVID-19 in patients with inflammatory arthritis (*n* = 103) in the US demonstrated that those receiving GCs had a higher risk of being admitted for COVID-19 infection (adjusted OR: 21.1; 95% CI: 4.1–109.0; *p* < 0.001), whereas patients on anticytokine bDMARDs did not [142]. Moreover, in the aforementioned publication from the Global Rheumatology Alliance Global Registry, a prednisone dose ≥10 mg/day was associated with a higher risk of hospitalisation with COVID-19 (OR: 2.1; 95% CI: 1.1–4.0) in patients with rheumatoid disease [103]. In the same study, the use of csDMARDs as a monotherapy or in combination with biologics or JAK inhibitors was not associated with an increased risk of hospitalisation (OR: 1.2, 95% CI: 0.7–2.2, and OR: 0.7, 95% CI: 0.4–1.5, respectively) [103]. Interestingly, TNF inhibitor use was associated with a reduced risk of hospitalisation (OR: 0.4; 95% CI: 0.2–0.8) [103]. Reassuringly, data from two small preliminary studies also indicate that patients receiving b/tsDMARD therapy do not appear to be at an increased risk of COVID-19-associated complications compared with the general population [100,143]. However, these studies are based on limited sample sizes. Results from the large meta-analysis by Akiyama et al. demonstrated that patients with autoimmune disease treated with GCs, csDMARDs or b/tsDMARD–csDMARD combination therapy had a 2–3 times higher rate of hospitalisation and death due to COVID-19 when compared with those treated with b/tsDMARD monotherapy [104]. Importantly, patients receiving TNF inhibitor monotherapy had a lower rate of hospitalisation and mortality compared with those receiving non-TNF-inhibitor monotherapy [104]. The association between rituximab treatment and COVID-19 risk is currently unknown; however, the incidence of severe COVID-19 infection was increased in patients treated with rituximab compared with patients treated with infliximab in a small single-centre study [144]. This finding was supported by two separate, small, single-centre studies that reported a higher risk of hospitalisation in patients treated with rituximab but not in those treated with TNF inhibitors or other bDMARDs [145,146]. Based on the current evidence, patients should be able to continue their current regimens, particularly if patients are receiving TNF inhibitor monotherapy. Preliminary guidance from EULAR advises that patients who do not have suspected or confirmed COVID-19 should continue their treatment regimen unchanged [140].

## 5. Lymphoma and NMSC in RA

### 5.1. Prevalence of Lymphoma in Patients with RA

Patients with RA have an increased risk of developing lymphoma, with a meta-analysis of eight epidemiological studies reporting that the risk is more than doubled compared with the general population (pooled standardised incidence ratio: 2.5) [147]. The risks of developing non-Hodgkin lymphoma (NHL) and Hodgkin lymphoma (HL) are both increased in patients with RA [147,148], with diffuse large B-cell lymphoma (DLBCL) the most frequently encountered lymphoma subtype [148,149]. A relatively recent study from Hellgren et al. in 2017 confirmed that the lymphoma risk in recently diagnosed RA patients has not decreased compared with that reported in historical cohorts, despite improvements in the management and treatment of RA [150]. This Swedish cohort study of 12,656 incident cases of RA reported a 60% increase in the risk of lymphoma compared with the population norm (HR: 1.6; 95% CI: 1.2–2.1). Because of this increased risk, it is recommended in a EULAR initiative that malignancies, such as lymphoma, are carefully assessed and managed in patients with chronic inflammatory rheumatic diseases [12].

### 5.2. Impact of RA on Outcomes in Patients with Lymphoma

A small number of studies have suggested that the presence of autoimmune diseases, including RA, may be an adverse prognostic factor among patients with lymphoma. For instance, a large cancer registry study in Sweden demonstrated that the risk of death due to cancer was significantly higher in patients with a previous hospitalisation for RA (*n* = 6309) than in patients without RA (*n* = 1,404,854) [151]. This was true for all cancers (HR for cause-specific survival: 1.3; 95% CI: 1.3–1.3) but especially for NHL (HR: 1.4; 95% CI: 1.2–1.7) and SCC (HR: 1.9; 95% CI: 1.0–3.5). A separate Swedish cohort study reported that a self-reported history of autoimmune disease (RA, systemic lupus erythematosus, Sjögren’s syndrome or coeliac disease; *n* = 97) was associated with an increased risk of all-cause death (HR: 1.4; 95% CI: 1.0–1.8; *p* = 0.03) and a trend for an increased risk of lymphoma-related death (HR: 1.3; 95% CI: 1.0–1.8; *p* = 0.08) in patients with NHL (*n* = 1523) [152]. More recently, a prospective cohort study by Kleinstern et al. demonstrated that a history of autoimmune conditions primarily mediated by B-cell responses was associated with worse event-free survival in patients with mantle cell lymphoma (HR: 2.2; 95% CI: 1.2–4.3; *n* = 193) or HL (HR: 2.6; 95% CI: 1.0–6.6; *n* = 297) and that this was largely driven by RA [153]. A hospital-based case–control study, also by Kleinstern et al., showed that in patients with B-cell NHL (*n* = 435), time to relapse was significantly shorter for patients with a history of autoimmune disease compared with control patients (HR: 1.7; 95% CI: 1.0–2.8) [154]. In patients with DLBCL (the most commonly observed B-cell NHL subtype), a history of B-cell-mediated autoimmune disease was associated with both shorter relapse-free survival (HR: 8.3; 95% CI: 3.0–23.1) and overall survival (HR: 3.8; 95% CI: 1.2–12.3) compared with controls [154]. In contrast, a smaller US study reported that NHL patients with RA (*n* = 65) had similar overall survival rates, but a significantly lower risk of lymphoma progression or relapse (HR: 0.4; 95% CI: 0.3–0.7) or death related to lymphoma or its treatment (HR: 0.6; 0.4–1.0), compared with NHL patients without RA (*n* = 1530) [155]. Similar results were reported when the analysis was limited to patients with DLBCL. Of note, NHL patients with RA were found to be twice as likely as patients without RA to die from causes unrelated to lymphoma or its treatment (HR: 2.2; 95% CI: 1.3–3.5) [155]. It is evident from the results of these studies that the association between RA and lymphoma outcomes is complex and requires further investigation.

### 5.3. Risk Factors for Development of Lymphoma in Patients with RA (Including Effect of TNF Inhibitors)

Factors that contribute to the development of lymphoma in patients with RA remain to be fully elucidated; however, there is strong evidence that the inflammatory processes underlying RA may also drive the development of lymphoma. This is supported by a number of studies, which were performed prior to the time period covered by our literature review, showing that the risk of lymphoma is higher in patients with more severe disease activity. For example, a nested case–control study of Swedish patients with RA prior to the introduction of biologics (1965–1983, *n* = 11 683) reported a strong association between high inflammatory disease activity and the risk of developing lymphoma (OR vs. low activity: 25.8; 95% CI: 3.1–213.0) [156]. Subsequently, in a matched case–control study of 378 patients with RA and lymphoma (1964–1995), the same group demonstrated that the risk of lymphoma was increased 70-fold in the subset of RA patients with the highest inflammatory disease activity (unadjusted OR vs. low activity: 71.3; 95% CI: 24.1–211.4), but by only eight-fold in patients with intermediate disease activity (unadjusted OR: 7.7; 95% CI: 4.8–12.3) [157]. A similar study of 1767 consecutive patients with RA with 15,832 patient-years of follow-up demonstrated that persistent inflammatory activity (defined as an elevated erythrocyte sedimentation rate) was associated with a nine-fold increase in the risk of NHL [158].

There is great interest in whether treatment with TNF inhibitors further increases the risk of lymphoma in patients with RA. Following the issuing of a US Food and Drug Administration (FDA) black box warning in 2009 for the use of TNF inhibitors and the risk of developing NHL (based on limited data), a nested case–control study within a retrospective cohort of adult patients with rheumatologic conditions (RA, PsA or AS), drawn from a US commercial health insurance database, was undertaken to fully assess any potential risk [159]. This analysis revealed that compared with matched controls (*n* = 984), patients with NHL (*n* = 101) had greater TNF inhibitor use (33% vs. 20%), with any use of a TNF inhibitor associated with an almost two-fold increased risk of NHL (OR: 1.9; 95% CI: 1.2–3.2). When analysed by drug, etanercept was associated with an increased NHL risk (OR: 2.7; 95% CI: 1.4–5.3), but no significant associations were reported for other TNF inhibitors. The findings from this study also suggested an increasing risk with increasing duration of therapy (*p*-value for trend: 0.05). Considering these observations, the authors concluded that the FDA black box warning was warranted and that continued surveillance for this potential adverse outcome is required [159]. In addition, a pooled analysis of 11,317 patients with autoimmune diseases treated with the TNF inhibitor certolizumab pegol in clinical trials demonstrated that for patients with RA, lymphatic and haematopoietic cancer incidence (principally lymphoma) was increased compared with the age- and gender-matched GLOBOCAN/SEER general population (standardised IR: 2.1; 95% CI 1.2–3.6) [160]. These results, however, differ from those from the BSRBR-RA. After adjusting for differences in baseline patient characteristics, a prospective cohort analysis of the BSRBR-RA database found no difference in the risk of lymphoma for RA patients receiving TNF inhibitors (*n* = 11,931) compared with those who were biologic-naïve (*n* = 3367) (HR: 1.0; 95% CI: 0.6–1.8), suggesting that TNF inhibition does not influence the risk of lymphoma over the background risk in patients with RA [161]. These results concur with the findings of a collaborative European registry study that examined the increased risk of both HL and NHL in patients with RA [149]. After examining the potential role of a variety of treatments, including bDMARDs and TNF inhibitors, the authors concluded that the risk of lymphoma in RA may be related to the disease itself rather than the chosen treatment approach, with use of TNF inhibitors having no apparent effect on the subtypes of lymphoma observed [149]. A study of 12,656 cases of incident RA from a Swedish registry also reported no impact of TNF inhibitors on the risk of lymphoma [150]. Moreover, a more recent study by the same Swedish group suggested no increase in lymphoma risk for patients with RA starting a first bDMARD (TNF inhibitor or non-TNF-inhibitor drug; *n* = 16,392) compared with bDMARD-naïve patients (*n* = 55,253) and no signals of different risk with any particular TNF inhibitor agent [162]. These accumulating data from large national registries, therefore, call into question the earlier data suggesting a link between TNF inhibitor use and the development of lymphoma. Notably, a recent overview of systematic reviews and meta-analyses of malignancy risk with TNF inhibitors noted no increase in lymphoma risk in patients with RA [163]. With respect to csDMARDs, there is currently no compelling evidence to suggest an association between methotrexate use and lymphoma risk in patients with RA, as reviewed in Klein et al. [148]. The aforementioned study of 12,656 cases of incident RA from a Swedish registry reported no impact of methotrexate on the risk of lymphoma [150]. However, a large historical case-control study of patients with RA from Canadian databases (*n* = 23,810) noted that the adjusted RR for lymphoma was slightly increased (1.2; 95% CI: 1.0–1.6) following methotrexate therapy [164].

### 5.4. Prevalence of NMSC in Patients with RA

Studies in patients with RA have reported increases in the risk of NMSC compared with the general population [165,166,167], including in those naïve to biologics [166,167]. These findings are supported by data from a large population-based cohort study in Sweden, which showed a small to moderately increased risk of BCC for biologic-naïve patients with RA (*n* = 46,409) compared with the general population (HR: 1.2; 95% CI: 1.1–1.4) [168]. The same study reported that the risk of SCC was nearly doubled in biologic-naïve patients compared with the general population (HR: 1.9; 95% CI: 1.7–2.0) [168].

### 5.5. Risk Factors for NMSC in Patients with RA

Studies have suggested a link between treatment, especially methotrexate and TNF inhibitors, and the risk of developing NMSC in patients with RA. A retrospective analysis of data from a mixed population of patients with RA or PsA in Tasmania, Australia (*n* = 405), identified an increased risk of both BCC and SCC among patients exposed to methotrexate [169]. Methotrexate use was associated with a standardised IR of 4.6 (95% CI: 0.7–33.2) for at least one confirmed NMSC case compared with no methotrexate usage. An increased risk of NMSC was also apparent among patients exposed to combinations of corticosteroids (RR: 2.5; 95% CI: 1.2–5.1) or D-penicillamine (RR: 3.5; 95% CI: 1.3–4.6), both with concomitant methotrexate. While not undertaken in RA patients, prespecified secondary analyses of the large Cardiovascular Inflammation Reduction Trial in 4786 adults with CVD and diabetes or metabolic syndrome reported an association between low-dose methotrexate use and skin cancers (HR: 2.1; 95% CI: 1.3–3.3), especially SCC (HR: 3.3; 95% CI: 1.6–6.7) [170]. These findings, which indicate a link between methotrexate use and the development of skin cancers in patients without inflammatory rheumatic conditions, could, in part, explain the higher rate of NMSC in patients with RA (most of whom have received methotrexate) compared with the general population [171].

The risk of NMSC in patients with RA receiving TNF inhibitors has been examined in multiple studies. In a recent systematic review and meta-analysis of six studies in 123,023 patients, an increased risk of NMSC (RR: 1.3; 95% CI: 1.2–1.4; *p =* 0.056), especially SCC (RR: 1.3; 95% CI: 1.1–1.5; *p* = 0.854) but not BCC (RR: 1.1; 95% CI: 1.0–1.3; *p* = 0.555), was observed in patients with RA treated with TNF inhibitors compared with patients not receiving TNF inhibitors [172]. A meta-analysis from Askling et al., comprising 74 RCTs using adalimumab, etanercept or infliximab (*n* = 15,418), also demonstrated that the overall risk of NMSC was doubled following TNF inhibitor use compared with comparator arms (HR: 2.0; 95% CI: 1.1–4.0) [173]. The incidence of NMSC was also evaluated in a national cohort of US veterans with RA receiving TNF inhibitors and non-bDMARDs (*n* = 20,648) [174]. Patients receiving TNF inhibitors had a higher risk of developing NMSC vs. patients receiving non-bDMARDs (HR: 1.4; 95% CI: 1.2–1.6) [174]. These findings suggest an increase in NMSC risk, particularly SCC risk, with TNF inhibitor therapy; further studies are required to explore the factors that underlie this increase.

## 6. Conclusions

Much of the literature implicates dysregulated systemic inflammatory responses in the pathogenesis of comorbidities associated with RA. While the contemporary clinical treatment goal is to achieve remission, the ideal immunological treatment goal is to achieve an immune homeostasis, such that the immune system can respond dynamically and appropriately to the environment. In keeping with this concept, the literature reviewed here emphasises the importance of optimal inflammation control, with a view to minimising or preventing the key complications of RA. However, immunosuppression, and a corresponding increase in infection risk, is a danger of over-treatment with TNF inhibitors and other targeted therapies. Similarly, the TNF blockade may be associated with an increased risk of NMSC, and, therefore, regular skin examination is desirable for patients with RA treated with TNF inhibitors. Conversely, TNF inhibitors and other biologics offer benefits that extend beyond amelioration of articular manifestations of RA and, in particular, to improvements in CV health. With the emergence of biosimilars to several TNF inhibitors, access to these agents is expected to rapidly increase in the coming years, potentially leading to initiation of treatments at an earlier disease stage. An anticipated advantage of earlier access to treatment, and a correspondingly improved control of systemic inflammation over time, is the prevention of CV complications that may otherwise be incurred due to sustained systemic inflammatory responses. Broader and earlier treatment with TNF inhibitors may also reduce the occurrence of other comorbidities as well as joint symptoms. However, it is important to mitigate the risk of overtreatment and immunosuppression, and this must be considered in the context of the responsible management of RA. For those patients achieving prolonged and sustained disease remission, step-down approaches may be appropriate, with a subsequent careful review of progress. A shared decision-making approach will be critical and should take into account the unique clinical situation of any given patient and the benefit–risk profile that they consider to be appropriate.

## Figures and Tables

**Table 1:** Prevalence of cardiovascular disease in patients with rheumatoid arthritis.

Citation	Country	Study Type	Patients (*n*)	CV Event	Prevalence (%)
Daniel 2020 [22]	USA	Systematic review	1,642,402	Atherosclerotic CVD	30%–47%
Panafinda 2013 [23]	Russia	Prospective, observational	200	Ischaemic heart disease MI Coronary artery bypass graft Stroke	19% 1.5% 3.5% 0.5%
Crowson 2017 [24]	International	Prospective, cohort	5638	CVD events *	Men: 20.9% ^†^ Women: 11.1% ^†^
Pappas 2018 [25]	International	Registry *CORRONA* International *CORRONA* US	25,987	Any CVD ^‡^	Latin America: 8.5% Eastern Europe: 21.3% India: 5.6% USA: 8.5%
Dougados 2014 [1]	International	Cross-sectional, observational *COMORA*	4586	MI or stroke	6.0%
Gomes 2017 [26]	Brazil	Cross-sectional, population-based	296	MI	4.4%
Lauper 2018 [27]	Switzerland	Mixed retrospective and prospective cohort	3070	MACE ^§^	2.67 per 1000 person-years ^¶^
Nikiphorou 2020 [19]	England	Retrospective, case–control	6591	MI, stroke or heart failure	10.62 per 1000 person-years
Agca 2020 [20]	Netherlands	Prospective cohort CARRÉ	326	CV event ^‖^	32 per 1000 person-years ^¶^
Solomon 2015 [28]	USA	Registry *CORRONA* US	24,989	MI, stroke or CV death	7.79 per 1000 person-years

* ACS including MI, stable or unstable angina, revascularisation, CV deaths, cerebrovascular events, peripheral vascular events. ^†^ Unadjusted cumulative incidence at 10 years after baseline. ^‡^ Including congestive heart failure, coronary artery disease, MI, unstable angina, peripheral arterial disease, stroke, transient ischaemic attack and other. ^§^ Defined as MI, transient or permanent cerebrovascular event, or CV-associated death. ^¶^ Unadjusted incidence rate. ^‖^ Defined as a verified medical history of coronary heart disease, cerebral arterial disease or peripheral arterial disease. Abbreviations: ACS, acute coronary syndrome; CARRÉ, CARdiovascular research and RhEumatoid arthritis; COMORA, COMOrbidities in Rheumatoid Arthritis; CORRONA, Consortium of Rheumatology Researchers of North America; CV, cardiovascular; CVD, cardiovascular disease; MACE, major adverse CV event; MI, myocardial infarction.

**Table 2:** Studies reporting an association between rheumatoid arthritis disease activity and risk of cardiovascular disease.

Citation	Study Type	Patients (*n*)	CV Event	Disease Activity Parameters with a Significant Impact on Risk of CVD	
				Parameter	Impact on Risk
Crowson 2017 [24]	Prospective, cohort	5638	Fatal or nonfatal CV events *	DAS28 RF/ACPA-positive	PAR: 12.6% PAR: 12.2%
Solomon 2015 [28]	Registry *CORRONA* US	24,989	Composite of MI, stroke or CV death	CDAI	Risk reduced by 21% per 10 pt reduction in time-averaged CDAI
Dalbeni 2020 [39]	Prospective	137	Ultrasound-detected atheromatous plaques	DAS28 (CRP) ≥ 2.6	Worsening of atherosclerosis only detected in patients with active disease
Arts 2017 [40]	Prospective, inception cohort	1157	Fatal or nonfatal CV events ^†^	DAS28 ≤ 3.2	Reduced risk of CVD (HR: 0.65; 95% CI: 0.43–0.99) ^‡^
Mantel 2015 [41]	Nested, case–control	138	ACS	Mean DAS28 EULAR ≥ 5.2 ^§^ ESR > 23/> 22 ^¶^ SJC > 6/> 4 ^¶^	OR: 1.32 (95% CI: 1.06–1.64) OR: 2.59 (95% CI: 1.04–6.43) OR: 3.01 (95% CI: 1.54–5.88) OR: 1.32 (95% CI: 1.06–1.64)
Ahlers 2020 [42]	Electronic health record analysis	6161	Heart failure	CRP	OR: 1.29 (95% CI: 1.16–1.44)
Bajraktari 2017 [43]	Cross-sectional	179	Hypertension	CRP, ESR, anti-CCP, DAS28	Significantly higher values reported in hypertensive patients (*p* < 0.001)
Berendsen 2017 [44]	Inception cohort	929	Fatal or nonfatal CV events ^‖^	RF positivity	HR: 1.52 (95% CI: 1.01–2.30) **

* ACS including MI, stable or unstable angina, revascularisation, CV deaths, cerebrovascular events, peripheral vascular events. ^†^ ACS, stable angina pectoris, cerebral vascular accident, transient ischaemic attack, peripheral artery disease and heart failure. ^‡^ With DAS28 as a time-dependent variable and after adjustment for confounders, including demographics and traditional CVD risk factors. ^§^ EULAR disease activity score. ^¶^ Upper tertiles of the population. ^‖^ Ischaemic heart disease, nonhaemorrhagic cerebrovascular accident or peripheral artery disease. ** After adjustment for confounders, including demographics and traditional risk factors. Abbreviations: ACPA, anticitrullinated protein antibodies; ACS, acute coronary syndrome; anti-CCP, anticyclic citrullinated peptide antibodies; CDAI, clinical disease activity index; CI, confidence interval; CORRONA, Consortium of Rheumatology Researchers of North America; CRP, C-reactive protein; CV, cardiovascular; CVD, cardiovascular disease; DAS28, Disease Activity Score, including 28 joints; ESR, erythrocyte sedimentation rate; EULAR, European League Against Rheumatism; HR, hazard ratio; MI, myocardial infarction; OR, odds ratio; PAR, population attributable risk; RF, rheumatoid factor; SJC, swollen joint count.

**Table 3:** Prevalence of infections in patients with rheumatoid arthritis.

Citation	Country	Study Type	Patients (*n*)	Infection Event	Prevalence, *n* (%) *
Doran 2002 [85]	USA	Retrospective, cohort	609	IRH	290 (47.6)
Mehta 2019 [88]	USA	Prospective, cohort	20,361	SI ^†^	1600 (7.9)
Ozen 2019 [89]	USA	Prospective, cohort	11,623	SI	694 (5.9)
Chandrashekara 2019 [90]	India	Cross-sectional	2081	Non-tubercular infection	54 (2.9)
Subesinghe 2016 [91]	UK	Cross-sectional	929	SI ^‡^	72 (7.8)
Salt 2017 [92]	USA	Retrospective, case–control	55,861	Postoperative joint infections ^§^	1127 (2.0)
Hashimoto 2017 [93]	Japan	Retrospective, single-centre	2688	IRH ^¶^	274 (10.2)
Rutherford 2018 [94]	UK	Prospective, cohort, registry: *BSRBR-RA*	19,282 ^‖^	SI **	5.51/100 patient-years
Richter 2016 [95]	Germany	Observational, cohort, registry: *RABBIT*	12,097	SI ^††^	947 (7.8)

* Unless otherwise stated. ^†^ SI including opportunistic and herpes zoster. ^‡^ At least one admission to secondary care with an infection in the preceding year. ^§^ In patients with RA or osteoarthritis following total joint arthroplasty. ^¶^ Including pneumocystis pneumonia. ^‖^ Patients with RA starting a new biologic. ** Defined as an infection resulting in death, hospitalisation or requiring intravenous antimicrobial therapy. ^††^ According to International Council for Harmonisation of Technical Requirements for Registration of Pharmaceuticals for Human Use definition. Abbreviations: BSRBR-RA, British Society for Rheumatology Biologics Register for Rheumatoid Arthritis; IRH, infection requiring hospitalisation; RA, rheumatoid arthritis; RABBIT, rheumatoid arthritis: observation of biologic therapy; SI, serious infection.

**Table 4:** Studies investigating associations between rheumatoid arthritis treatment regimens and the risk of infection.

Citation	Study Type	Patients (*n*) *	Infection Event	Treatments with an Impact on the Risk of Infection	
				Treatment	Impact on Risk
Hashimoto 2017 [93]	Retrospective, single-centre	342	IRH	GC	OR (95% CI): 3.0 (2.1–4.4); *p* < 0.0001 (2 mg/day)
				bDMARD	OR (95% CI): 1.4 (1.0–2.0); *p* = 0.033
				MTX	OR (95% CI): 0.7 (0.6–1.0); *p* = 0.034
Mehta 2019 [88]	Prospective, cohort	20,361	SI	GCs	HR (95% CI) for patients with RA vs. NIRMD: Excluding GCs: 1.7 (1.5–1.9) Adjusted for GCs: 1.3 (1.2–1.5)
Richter 2016 [95]	Observational, cohort, registry: *RABBIT*	1017	Sepsis and mortality following SI	GCs	OR (95% CI) for sepsis: GC 5 to <10 mg/day vs. ref ^†^: 1.3 (0.8–1.9) GC ≥ 10 mg/day vs. ref ^†^: 1.7 (1.0–2.9) OR (95% CI) for death: GC 5 to <10 mg/day vs. ref ^†^: 0.9 (0.5–1.8) GC ≥ 10 mg/day vs. ref ^†^: 2.4 (1.0–5.6)
				TNF inhibitor	OR (95% CI) sepsis vs. ref ^‡^: 0.6 (0.4–1.0) OR (95% CI) death vs. ref ^‡^: 0.5 (0.2–1.0)
				Other bDMARD	OR (95% CI) sepsis vs. ref ^‡^: 0.5 (0.3–0.8) OR (95% CI) death vs. ref ^‡^: 0.2 (0.1–0.5)
Ozen 2019 [89]	Prospective, cohort	11,623	SI	TNFis	Adjusted HR (95% CI) vs. ref ^‡^: 1.3 (1.1–1.7)
				Non-TNFi bDMARD	Adjusted HR (95% CI) vs. ref ^‡^: 1.5 (1.0–2.2)
Strand 2015 [115]	Meta-analysis	66 RCTs; 22 LTS	SI	Tofacitinib 5 mg BID Tofacitinib 10 mg BID Abatacept TNFis Rituximab Tocilizumab	Risk differences (95% CI) vs. placebo ^§^: 0.4% (−0.2–1.0) 0.4% (−0.2–1.0) 0.4% (−0.7–1.5) 0.9% (0.3–1.6) −0.4% (−1.6−0.8) 1.5% (0.7–2.3)
Singh 2015 [116]	Meta-analysis	106 studies (*n* = 42,330)	SI	bDMARD: low dose; standard dose; high dose	OR (95% CI) vs. ref ^‡^: 0.9 (0.7–1.3); 1.3 (1.1–1.6); 1.9 (1.5–2.4)
Minozzi 2016 [117]	Meta-analysis	71 RCTs; (*n* = 22,720); 7 OLE (*n* = 2236) ^¶^	SI	TNFis vs. placebo	Fixed-effects model (OR: 1.4; 95% CI: 1.2–1.7) Random-effects model (OR: 1.3; 95% CI: 1.0–1.6)

* Unless otherwise stated. ^†^ Reference; GC (<5 mg/day). ^‡^ Reference; csDMARD. ^§^ DMARD-inadequate responder patients only. ^¶^ With RA, PsA or AS. Abbreviations: AS, ankylosing spondylitis; bDMARD, biologic DMARD; BID, twice daily; CI, confidence interval; csDMARD, conventional synthetic DMARD; DMARD, disease-modifying antirheumatic drug; GCs, glucocorticoids; HR, hazard ratio; IRH, infection requiring hospitalisation; LTS, long-term extension study; MTX, methotrexate; NIRMD, noninflammatory rheumatic and musculoskeletal diseases; OLE, open-label extension study; OR, odds ratio; PsA, psoriatic arthritis; RA, rheumatoid arthritis; RABBIT, Rheumatoid Arthritis: Observation of Biologic Therapy; RCT, randomised controlled trial; Ref, reference; SI, serious infection; TNFi, tumour necrosis factor-α inhibitor.

## Supplementary Materials

The following are available online at https://www.mdpi.com/2077-0383/10/3/509/s1. Table S1: PubMed search strings.
