# Supplementary material for: The Key Comorbidities in Patients with Rheumatoid Arthritis: A Narrative Review
Source: J Clin Med. 2021 Feb 1;10(3):509. doi: 10.3390/jcm10030509 (PMC7867048; doi:10.3390/jcm10030509)
Supplement: Supplementary file 1 [file jcm-10-00509-s001.pdf]

Journal of
Clinical Medicine

MDPI

Supplementary Materials

Table S1. PubMed search strings.

**Search number**	**Query**	**Results**
2	(((arthritis, rheumatoid[MeSH Major Topic]) OR (rheumatoid arthritis[Title])))	16 739
3	((cardiovascular[Title] OR heart[Title] OR hypertension[Title] OR atrial[Title] OR coronary[Title]))	163 368
4	((infection[Title] OR infections[Title]))	91 892
5	((lymphoma[Title] OR lymphomas[Title]))	21 457
6	((((squamous cell carcinoma[Title] OR squamous cell carcinomas[Title] OR SCC[Title]))) OR ((basal cell carcinoma[Title] OR basal cell carcinomas[Title] OR BCC[Title]))) AND ("non-melanoma skin cancer"[Title]) OR "non melanoma skin cancer"[Title])	210
7	(((prevalence[MeSH Major Topic]) OR (prevalence[Title])))	40 667
8	(((((risk factors[MeSH Major Topic]) OR (pathology[MeSH Major Topic])) OR (inflammation[MeSH Major Topic])) OR (risk factor[Title] OR risk factors[Title] OR pathology[Title] OR pathogenesis[Title] OR etiology[Title] OR inflammation[Title] OR inflammatory[Title] OR cause[Title] OR causes[Title] OR screening[Title])))	230 721
9	(((((((((((quality of life[MeSH Major Topic]) OR (pain[MeSH Major Topic])) OR (patient reported outcome measures[MeSH Major Topic])) OR (outcome assessment, health care[MeSH Major Topic])) OR (treatment outcome[MeSH Major Topic])) OR (fatal outcome[MeSH Major Topic])) OR (disability evaluation[MeSH Major Topic])) OR (economics, medical[MeSH Major Topic])) OR (medication adherence[MeSH Major Topic])) OR ("quality of life"[Title] OR "life quality"[Title] OR "outcome"[Title] OR "outcomes"[Title] OR "fatal"[Title] OR "death"[Title] OR "survival"[Title] OR "burden"[Title] OR "economic"[Title] OR "cost"[Title] OR "adherence"[Title] OR "persistence"[Title] OR "compliance"[Title] OR "management"[Title] OR "decision"[Title] OR "decisions"[Title] OR "impact"[Title] OR "impacts"[Title] OR "effect"[Title] OR "effects"[Title] OR "influence"[Title] OR "influences"[Title])))	878 306
10	(((tumor necrosis factor inhibitors[MeSH Major Topic]) OR (tumour necrosis factor[tiab] OR tumour necrosis factors[Title] OR tumor necrosis factor[Title] OR tumor necrosis factors[Title] OR TNF[Title] OR adalimumab[Title] OR certolizumab[Title] OR golimumab[Title] OR etanercept[Title] OR infliximab[Title] OR biologic[Title] OR bDMARD[Title])))	16 825
11	((#2) AND (#3 OR #4 OR #5 OR #6)) AND (#7 OR #8 OR #9 OR #10)	**335**

Publisher's Note: MDPI stays neutral with regard to jurisdictional claims in published maps and institutional affiliations.

© 2020 by the authors. Submitted for possible open access publication under the terms and conditions of the Creative Commons Attribution (CC BY) license (http://creativecommons.org/licenses/by/4.0/).
